# Knowledge, attitudes, and practices regarding *Helicobacter pylori*-induced gastric ulcers and cancers among Saudi residents: a nationwide web-based survey

**DOI:** 10.3389/fpubh.2026.1904953

**Published:** 2026-09-04

**Authors:** Mohammed A. Almalki, Mohammed E. Alzhrani, Rayan T. Alzahrani, Sultan A. Alqahtani, Mazen I. Aljuaid, Azza A. K. El-Sheikh, Yassmin M. Shebany, Sayed F. Abdelwahab

**Affiliations:** 1 College of Pharmacy, Taif University, Taif, Saudi Arabia; 2 Basic Health Sciences Department, College of Medicine, Princess Nourah Bint Abdulrahman University, Riyadh, Saudi Arabia; 3 Biology Department, Faculty of Science, Taif University, Taif, Saudi Arabia; 4 Pharmaceutics and Industrial Pharmacy Department, College of Pharmacy, Taif University, Taif, Saudi Arabia

**Keywords:** attitudes, gastric cancer, gastric ulcer, *Helicobacter pylori*, knowledge, practice, Saudi Arabia

## Abstract

**Background and aim:**

*Helicobacter pylori (H. pylori)* is one of the most common worldwide infections that represent a significant public health problem due to its strong connection with chronic gastritis, peptic ulcers, and gastric cancers. Few studies have looked at the public’s knowledge, attitudes, and practices (KAP) regarding *H. pylori* infection and its impact, both globally and in Saudi Arabia. The purpose of this study was to evaluate Saudi residents’ KAP concerning *H. pylori*-induced gastric ulcers and malignancies, and to determine the sociodemographic variables affecting awareness and preventive actions.

**Methods:**

The study was conducted using a cross-sectional descriptive design, focusing on Saudi residents in different locations who are at least 18 years old. Informed consent was obtained, and ethical approval of the study was granted by Taif University. An online self-administered questionnaire that underwent expert validation and a pilot study to ensure clarity and reliability was distributed via social media platforms to collect data. The IBM SPSS version 25 was used for statistical analyses, applying both descriptive and inferential approaches at a significance level of *p* ≤ 0.05.

**Results:**

A total of 1,482 participants were included in this study. The findings indicated low knowledge and moderate attitude and practice levels, with mean scores of 55.5, 64.2, and 66.7% for knowledge, attitudes and practices, respectively. Several knowledge gaps were identified, particularly regarding treatment, where a considerable proportion of the participants incorrectly believed that antibiotics or antacids alone are sufficient for treatment (48.4 and 45.3%, respectively). Participants generally demonstrated positive attitudes, while practices showed some inappropriate behaviors, including self-medication and early discontinuation of antibiotics. Significant associations were observed between KAP domains and several sociodemographic factors.

**Conclusion:**

This study underscores the need to improve the KAP regarding *H. pylori* infection among Saudi residents, as the overall KAP levels were low or moderate emphasizing the need for targeted and effective public health interventions to reduce the burden of *H. pylori*–related diseases.

## 1 Introduction

*Helicobacter pylori (H. pylori)* is a Gram-negative, spiral-shaped bacterium that inhabits the gastric mucosa. It is one of the most common bacterial infections in the world, the prevalence of which is estimated to be more than half of the global population (1, 2). The main route of transmission is the fecal-oral route, which is mainly through the consumption of contaminated food and/or water or through direct contact with saliva or other body fluids of infected hosts (1). Although many individuals with *H. pylori* infection do not develop symptoms, the presence of the bacterium in the gastric mucosa may lead to gastritis or peptic ulcer disease. Symptoms of peptic ulcer may include dull abdominal pain that can occur several hours after meals, appear during the night when the stomach is empty, or comes and goes over days or weeks. The pain may improve temporarily with food intake or antacid use. Other symptoms associated with peptic ulcer disease include weight loss, reduced appetite, abdominal bloating, frequent burping, nausea, vomiting, and anemia resulting from gastrointestinal bleeding (2). The nature of *H. pylori* presence in the gastric mucosa predisposes individuals to chronic gastritis and peptic ulcer disease and is a well-established risk factor for gastric adenocarcinoma, the most serious complication of the infection. Infection rates are reported to be higher in developing regions, where overcrowding, poor sanitation, and limited access to clean water increase the risk of transmission (1, 3). Some lifestyle risk factors, including nutrition and tobacco consumption, could also increase vulnerability to infection and infection-related complications (1). Combination regimens of antimicrobial agents together with acid-suppressive drugs are effective in eradicating *H. pylori* when used as prescribed (2). However, the occurrence of reinfection and the development of antibiotic-resistant strains of bacteria is one of the greatest issues worldwide, which can be explained by the fact that not all people follow the treatment regimen and are not aware of the risks of infection and preventive actions (1, 3).

Several studies have examined the levels of knowledge, attitudes and practices (KAP) toward *H. pylori* infection. These studies showed a consistent lack of awareness among different populations. Among these, a cross-sectional survey of 808 adult *patients* with a clinical diagnosis of *H. pylori* infection in the Riyadh region revealed that over 50 % of the respondents involved had good knowledge of the pathogen, though almost half of them were not aware of the relation between the pathogen and gastric cancer. The authors concluded that the level of general awareness was acceptable, but the presence of misconceptions about the risk of cancer remained, which explains the need to conduct educational interventions focused on the elimination of these misconceptions (4). Another study conducted at King Saud University in which 334 *undergraduate students* in Riyadh (189 health-related, 145 non-health disciplines) were assessed for *H. pylori* infection knowledge. Although most of them had previously heard about the organism, fewer than 10% of them showed sufficient knowledge about its methods of transmission, complications, or prevention. Misconceptions were, also, prevalent in both groups (5). Also, a descriptive cross-sectional survey of 330 *medical students* attending King Saud bin Abdulaziz University for Health Sciences across different years of study was conducted. Findings revealed that 42% of the participants had average knowledge, 41% had excellent knowledge, 17% had poor knowledge, and still lacked information on the transmission routes and preventive measures. It is worth noting that male students in the clinical years (fifth and sixth) had significantly higher awareness (*p* < 0.001) (6). Importantly, a cross-sectional national study of 474 *physicians* of various specialties was conducted to determine the level of knowledge and perception towards *H. pylori* infection. The sample was dominated by males (67.3%) and the largest subgroup was internal medicine specialists (30%). The overall level of awareness (76.9 ± 6.9%) was found to be adequate, though it was also found that awareness was significantly associated with age, years of experience, hospital sector, and region of practice, with physicians in the southern and central regions having higher scores (7).

To pinpoint international patterns of understanding of *H. pylori* and its determinants, researchers in the broader region and internationally have studied public and professional knowledge about *H. pylori*. A descriptive cross-sectional survey of 767 *adults* in different regions of *Jordan* showed that 68.4% of the participants had poor attitudes and practices regarding *H. pylori* infection. The authors concluded that the level of public awareness and behavioral practices were not satisfactory (8). Another cross-sectional study of 398 adults in Jordan found that 64.3% had heard of *H. pylori* and 75.9% recognized its association with gastric ulcers, although important gaps remained regarding transmission and complications. Employment status was significantly associated with knowledge, with medical participants demonstrating higher knowledge than non-medical participants (9). Also, in Samsun, *Turkey*, general familiarity with the organism was observed in a cross-sectional survey of 109 *general practitioners* with significant gaps in diagnostic methods and overall management (10). At the international level, knowledge and clinical practices regarding the diagnosis and treatment of *H. pylori* infection among *primary care physicians* were evaluated in a hospital-based cross-sectional study of 382 physicians across four regions in *Cameroon*. More than 80% of the participants incorrectly believed that *H. pylori* causes gastroesophageal reflux disease. Differences in diagnostic and treatment practices were observed among physicians of different regions. The authors concluded that there was insufficient overall knowledge and compliance with international recommendations (11). Another cross-sectional survey of 1,042 *adults* across 49 *American States* investigated the public awareness, experiences, and barriers related to *H. pylori* infection and treatment. About 52.3% had heard about *H. pylori*, and 11.7% had previously received treatment. Of those treated, 96.7% reported difficulties completing the therapeutic course, mainly due to gastrointestinal side effects and cost. Reported barriers to healthcare access included limited financial resources, lack of insurance, and scheduling difficulties. Overall awareness about *H. pylori* was insufficient, and treatment adherence remained one of the primary challenges (12). In Japan, a nationwide survey of 3,095 adults found high awareness of *H. pylori* (89.1%), but limited understanding of its characteristics (56.4%), with an overall knowledge accuracy of 45.7% and particularly low knowledge regarding testing and treatment (37.5%) (13). Similarly, a cross-sectional study of 712 residents in Northeast China found limited knowledge (mean score = 53.8%) despite positive attitudes (81.8%) and preventive practices (68%), with social media use significantly associated with higher knowledge, more positive attitudes, and better preventive practices (14).

Overall, the above-mentioned studies highlight the need for comprehensive educational initiatives targeting the general population both globally and locally at the Kingdom. The local studies (4–7) are limited by low sample size, regions studied, and by providing only some insight about *H. pylori*-related KAP levels among specific populations in certain localities of the Kingdom and suggest a scarcity of surveys of the general Saudi population at the national level. To our knowledge, no previous nationwide study has comprehensively assessed the KAP levels regarding *H. pylori*-associated gastric ulcers and gastric cancer among the general Saudi population. Understanding these issues is essential, as they influence early detection, adherence to treatment, and prevention of complications. Given the global burden of *H. pylori* infection and its established role in gastric carcinogenesis, this study examined the *H. pylori*- infection related KAP among Saudi residents to identify the sociodemographic determinants influencing awareness, preventive behaviors, and health-seeking patterns. We identified low knowledge and moderate levels of attitudes and practices regarding *H. pylori* infection among Saudi residents, with treatment-related knowledge representing the most consistently limited domain. Item-level findings indicated that most of the participants incorrectly responded to or expressed uncertainty about the components of eradication therapy, while basic preventive knowledge was comparatively more established. Selected sociodemographic variables were associated with performance on specific KAP items, though these associations were variable across all domains. The findings of this study provide a comprehensive baseline data on public KAP towards *H. pylori*-associated gastric ulcers and gastric cancer among Saudi residents. This may contribute to national health education strategies that enhance public awareness and promote preventive behaviors that may reduce the incidence of *H. pylori* infection and its complications in the Kingdom of Saudi Arabia.

## 2 Methods

### 2.1 Study design

This was a cross-sectional descriptive study to assess the KAP levels among Saudi residents regarding *H. pylori*-induced gastric ulcers and cancers. This design is appropriate for evaluating awareness levels and behavioral patterns among a defined population at a single point in time.

### 2.2 Questionnaire development and validation

Data were collected using a structured, self-administered questionnaire developed in accordance with the World Health Organization (WHO) KAP survey framework. The instrument was adapted from previously validated tools published in peer-reviewed studies focusing on awareness of *H. pylori* infection, transmission, and prevention (14–19). The questionnaire consisted of four main sections with a total of 54 items, distributed as follows:

Section 1: Demographic information (nine items): covering age, gender, region of residence, education level, occupation, income, and other relevant sociodemographic factors.

Section 2: Knowledge (15 questions): assessing understanding of *H. pylori* transmission routes, risk factors, symptoms, diagnostic methods, treatment, and its link to gastric ulcers and cancers.

Section 3: Attitudes (15 items): exploring perceptions toward *H. pylori* infection, preventive measures, willingness to undergo screening, and confidence in healthcare provider’s advice.

Section 4: Practices (15 items): evaluating personal hygiene, dietary habits, medical consultation behavior, and treatment adherence.

The questionnaire was prepared in both Arabic and English to ensure clarity and accessibility for all participants. It was reviewed by a panel of four experts to evaluate its content validity, clarity, and cultural appropriateness for the target population. Based on the experts’ recommendations, minor modifications were made to improve the wording, clarity, and overall comprehensibility of several items before pilot testing. Subsequently, a pilot study was conducted among 28 participants to assess the clarity, comprehensibility, and reliability of the questionnaire. Data obtained during the pilot study were not included in the final analyses. Importantly, validity and internal consistency of the questionnaire were confirmed using Cronbach’s alpha analysis for the knowledge, attitude and practice sections with alpha values of 0.73, 0.92 and 0.84, respectively.

### 2.3 Ethical considerations

Ethical approval of the study was obtained from the Institutional Review Board (IRB) at Taif University before commencing the study (approval # 47–117). Participation was voluntary, and informed consent was obtained electronically before completing the online questionnaire. All responses remained anonymous and confidential, and no personal identifiers were collected. The study adhered to the ethical principles defined in the 1964 Helsinki Declaration.

### 2.4 Sample size and sampling technique

The study targeted adults aged 18 years and older residing in various Saudi Arabian regions. The minimum sample size was calculated using the Raosoft sample size calculator with 95% confidence level, 5% margin of error, and 50% response distribution, yielding a minimum of 385 participants. A non-probability convenience sampling method was utilized to recruit participants via online and social media platforms.

### 2.5 Data collection

Data were collected electronically through a Google Form. To prevent duplicate submissions, responses were limited to a single response per Google user/email by adjusting the Google Form settings. The questionnaire link was distributed across popular social media platforms (e.g., WhatsApp, X (Twitter), Instagram, and Telegram) to maximize participants’ reach. Respondents were required to read and agree on an informed consent statement before participation. Data were automatically recorded and stored securely in a password-protected database accessible only to the research team. A total of 1,488 responses were received through the online survey. Of these 1,488 respondents, six participants (0.4%) declined to provide electronic informed consent and were excluded while 1,482 participants (99.6%) provided this consent and were included in the final analysis.

### 2.6 Calculations of KAP scores

The KAP scores were calculated based on the participants’ responses to the structured questionnaire. All items were close ended. Knowledge items used “Yes, No and I don’t know” responses. For the knowledge domain, each correct response was assigned a score of one point while the incorrect or “I don’t know” responses were assigned a zero score. The total knowledge score was obtained by summing the correct responses and converting it into a percentage of the maximum possible 100% score.

For the attitude domain, responses were measured using a five-point Likert scale (strongly agree, agree, neutral, disagree, strongly disagree), which were assigned scores from 5 to 1, respectively. Higher scores indicated a more positive attitude. Negatively worded attitude items were reverse scored prior to analyses. The total attitude score was calculated by summing the item scores and converting it into a percentage of the maximum possible score.

For the practice section, responses were assessed using a five-point Likert scale (never, rarely, sometimes, often and always), which were assigned scores from 1 to 5, respectively, with higher scores indicating better practices. Negatively worded practices were reverse scored prior to analyses. The total practice score was calculated by summing the responses and converting it into a percentage of the maximum possible score. For the general interpretation of the KAP scores, the percentages were categorized according to the modified Bloom’s cut-off criteria as low/poor (<60%), moderate (60–79%), and good (≥80%).

### 2.7 Data analysis

Collected data were analyzed using International Business Machines Corporation Statistical Package for the Social Sciences (IBM-SPSS version 25). As all questionnaire items were mandatory in the Google survey, the final dataset contained no missing data. Descriptive statistics-including frequencies, percentages, means, and standard deviations-were used to summarize demographic characteristics and KAP levels. Inferential statistical analyses were performed to explore associations between independent variables (e.g., demographics) and KAP items. The Chi-square test (*χ*^2^) was used for categorical variables. It should be noted that no corrections were applied for multiple comparisons (e.g., Bonferroni or FDR methods), despite the large number of chi-square tests performed in the three KAP domains. A *p*-value ≤ 0.05 was considered statistically significant.

## 3 Results

### 3.1 Demographic characteristics of the study participants

Table 1 shows the sociodemographic characteristics of the study participants. The study included 1,482 participants, with males representing a slightly higher proportion (55.7%, *n* = 825) compared to females (44.3%, *n* = 657). Regarding age distribution, participants aged 18–30 years constituted the most frequent group (33.4%, *n* = 495), followed by those aged 41–50 years (25.8%, *n* = 383). Most of the participants were married (58.7%, *n* = 870), while single participants accounted for only 37.7% (*n* = 558). Most participants were Saudi nationals (92.1%, *n* = 1,365), and the Western region represented the highest proportion (47.7%, *n* = 707), followed by the Central region (21.3%, *n* = 316). In terms of employment status, government employees represented the largest group (39.5%, *n* = 585), followed by students or unemployed individuals (30.7%, *n* = 455). Most of the participants held a bachelor’s degree (48.2%, *n* = 715), while 32.6% (*n* = 483) had a high school or diploma qualifications. Regarding monthly income, the highest proportion of the participants reported earning >10,000 SAR (40.1%, *n* = 594), followed by those earning <5,000 SAR (30.4%, *n* = 450). Finally, most participants were non-smokers (82.2%, *n* = 1,218), with smokers and ex-smokers representing smaller proportions (Table 1).

**Table 1:** Sociodemographic characteristics of the study participants.

Variable	Category	Frequency *n* (%)
Gender	Female	657 (44.3%)
	Male	825 (55.7%)
Age (years)	18–30	495 (33.4%)
	31–40	255 (17.2%)
	41–50	383 (25.8%)
	51–60	277 (18.7%)
	61 and above	72 (4.9%)
Marital status	Single	558 (37.7%)
	Married	870 (58.7%)
	Divorced	21 (1.4%)
	Widowed	33 (2.2%)
Nationality	Saudi	1,365 (92.1%)
	Non-Saudi	117 (7.9%)
Region of residence	Western Region	707 (47.7%)
	Central Region	316 (21.3%)
	Southern Region	220 (14.8%)
	Eastern Region	130 (8.8%)
	Northern Region	109 (7.4%)
Employment status	Government employee (full-time)	585 (39.5%)
	Student/Unemployed	455 (30.7%)
	Private sector employee (full-time)	165 (11.1%)
	Retired/Volunteer	137 (9.2%)
	Employed (part-time)	75 (5.1%)
	Self-employed	65 (4.4%)
Highest level of education	Less than high school	67 (4.5%)
	High school or Diploma	483 (32.6%)
	Bachelor’s degree	715 (48.2%)
	Higher diploma degree	103 (7.0%)
	Master’s degree or Doctorate (PhD)	114 (7.7%)
Average monthly income	Less than 5,000 SAR	450 (30.4%)
	5,000–10,000 SAR	438 (29.6%)
	More than 10,000 SAR	594 (40.1%)
Smoking status	Non-smoker (never smoked)	1,218 (82.2%)
	Regular smoker (daily/continuous)	107 (7.2%)
	Ex-smoker (quit smoking)	88 (5.9%)
	Social smoker (occasional)	69 (4.7%)

### 3.2 Assessment of the participants’ knowledge toward *H. pylori* infection

Table 2 presents the participants’ knowledge regarding *H. pylori* infection. The knowledge score was calculated as described in the Methods section and the average knowledge score was 55.5%, indicating a low level of awareness. The knowledge score for individual knowledge questions is shown on the far-right column. In this regard, half of the participants correctly identified bacteria as the cause of *H. pylori* infection (50.1%, *n* = 743). Regarding transmission through contaminated food or water, 64.8% (*n* = 960) of the participants answered correctly. Regarding treatment, only 30.8% (*n* = 457) of the participants answered correctly.

**Table 2:** Frequency distribution of participants’ knowledge toward *H. pylori* infection.

Knowledge statement	Response	Frequency *n* (%)	Knowledge score
*H. pylori* infection is caused by bacterial infection	Yes	743 (50.1%)	(50.1%)
	No	372 (25.1%)	
	I do not know	367 (24.8%)	
*H. pylori* can be transmitted through contaminated food or water	Yes	960 (64.8%)	(64.8%)
	No	257 (17.3%)	
	I do not know	265 (17.9%)	
Antibiotics alone are sufficient to treat *H. pylori* infection	Yes	718 (48.4%)	(30.8%)
	No	457 (30.8%)	
	I do not know	307 (20.7%)	
*H. pylori* infection can cause peptic ulcer disease	Yes	926 (62.5%)	(62.5%)
	No	277 (18.7%)	
	I do not know	279 (18.8%)	
Drinking unsafe or untreated water can cause *H. pylori* infection	Yes	904 (61.0%)	(61.0%)
	No	305 (20.6%)	
	I do not know	273 (18.4%)	
*H. pylori* infection is treated by antacids only	Yes	671 (45.3%)	(29.4%)
	No	435 (29.4%)	
	I do not know	376 (25.4%)	
*H. pylori* infection can be diagnosed using non-invasive tests such as the urea breath test or stool antigen test	Yes	914 (61.7%)	(61.7%)
	No	296 (20.0%)	
	I do not know	272 (18.3%)	
Washing hands before eating helps prevent *H. pylori* infection	Yes	991 (66.9%)	(66.9%)
	No	292 (19.7%)	
	I do not know	199 (13.4%)	
Living with someone who has *H. pylori* increases the risk of infection	Yes	730 (49.3%)	(49.3%)
	No	452 (30.5%)	
	I do not know	300 (20.2%)	
A common symptom of *H. pylori* infection is heartburn or pain in the upper abdomen	Yes	933 (63.0%)	(63.0%)
	No	298 (20.1%)	
	I do not know	251 (16.9%)	
Long-term *H. pylori* infection can lead to stomach (gastric) cancer	Yes	782 (52.8%)	(52.8%)
	No	291 (19.6%)	
	I do not know	409 (27.6%)	
The breath test is one of the most accurate tests for diagnosing *H. pylori* infection	Yes	790 (53.3%)	(53.3%)
	No	359 (24.2%)	
	I do not know	333 (22.5%)	
Completing the full course of treatment is important for *H. pylori* eradication	Yes	946 (63.8%)	(63.8%)
	No	291 (19.6%)	
	I do not know	245 (16.5%)	
Consuming fast food frequently may increase the risk of *H. pylori* infection	Yes	884 (59.6%)	(59.6%)
	No	287 (19.4%)	
	I do not know	311 (21.0%)	
Washing fruits and vegetables well helps prevent *H. pylori* infection	Yes	942 (63.6%)	(63.6%)
	No	295 (19.9%)	
	I do not know	245 (16.5%)	
Mean knowledge score	55.5%		

Regarding complications of the infection, the survey showed that 62.5% of the participants (*n* = 926) reported that *H. pylori* infection can cause peptic ulcer disease. For transmission through unsafe or untreated water, 61.0% (*n* = 904) of the participants answered correctly. Similarly, 45.3% of the participants (*n* = 671) incorrectly believed that antacids alone can treat the infection and only 29.4% (*n* = 435) responded correctly (Table 2).

Regarding diagnostic methods, 61.7% of the participants (*n* = 914) correctly identified non-invasive tests such as the urea breath test or stool antigen test can be used for diagnosis of *H. pylori* infection. In terms of preventive practices, about two thirds (66.9%; *n* = 991) of the participants identified hand washing as a preventive measure. With respect to risk factors, almost half of the participants (49.3%; *n* = 730) reported that living with an infected individual increases the risk of infection. For *H. pylori* symptoms, 63.0% (*n* = 933) of the participants identified heartburn or upper abdominal pain as common symptoms of the infection while the remaining did not. Also, only 52.8% of the participants (*n* = 782) recognized association of *H. pylori* infection with gastric cancer. In addition, the urea breath test was identified as one of the most accurate diagnostic tools by only 53.3% of the participants (*n* = 790; Table 2). Moreover, 63.8% of the participants (*n* = 946) recognized the importance of completing the full treatment course. Furthermore, 59.6% of the participants (*n* = 884) believed that frequent consumption of fast food may increase the risk of infection. Finally, washing fruits and vegetables was identified as a preventive measure by 63.6% of the participants (*n* = 942; Table 2).

### 3.3 Assessment of the participants’ attitudes toward *H. pylori* infection

Table 3 presents the distribution of participants’ attitudes toward *H. pylori* infection and its prevention. The attitude score of the participants was calculated as described in the Methods section and the average attitude score was 64.2%, indicating a moderately positive attitude. The individual attitude scores are shown on the far-right column. For the perception of *H. pylori* infection as a public health issue in Saudi Arabia, only 15.3% of the participants (*n* = 227) strongly agreed/agreed. In terms of the importance of prevention, less than half of the participants strongly disagreed/disagreed with the statement that preventing *H. pylori* infection is not important (*n* = 597, 40.3%), while a comparable proportion strongly agreed/agreed (*n* = 613, 41.4%). Concerning the seriousness of the infection, more than half of the participants (*n* = 818, 55.2%) strongly agreed/agreed that *H. pylori* infection is a serious health issue requiring professional medical attention. Regarding screening for *H. pylori*, about one third of the participants (*n* = 487, 32.9%) strongly disagreed/disagreed with the statement that regular screening is unnecessary. With respect to *H. pylori* preventive attitudes, more than half of the participants (*n* = 827, 55.8%) strongly agreed/agreed that maintaining proper hand hygiene and safe food practices can prevent infection, while less than a quarter of them strongly disagreed/disagreed (*n* = 344, 23.2%; Table 3).

**Table 3:** Frequency distribution of participants’ attitude toward *H. pylori* infection.

Attitude statement	Strongly disagree	Disagree	Neutral	Agree	Strongly Agree	Attitude score
*H. pylori* infection is a major public health issue in Saudi Arabia	642 (43.3%)	295 (19.9%)	318 (21.5%)	153 (10.3%)	74 (5.0%)	42.8
Preventing *H. pylori* infection is not important for maintaining good digestive health	342 (23.1%)	255 (17.2%)	272 (18.4%)	407 (27.5%)	206 (13.9%)	61.6
*H. pylori* infection is a serious health issue that requires professional medical attention	146 (9.9%)	172 (11.6%)	346 (23.3%)	530 (35.8%)	288 (19.4%)	68.7
Regular screening for *H. pylori* is unnecessary and does not contribute to the prevention of gastric cancer	247 (16.7%)	240 (16.2%)	338 (22.8%)	460 (31.0%)	197 (13.3%)	58.4
Maintaining good hand hygiene and safe food practices can prevent *H. pylori* infection	162 (10.9%)	182 (12.3%)	311 (21.0%)	509 (34.3%)	318 (21.5%)	68.6
*H. pylori* infection can be completely cured when the correct treatment is followed	137 (9.2%)	178 (12.0%)	327 (22.1%)	515 (34.8%)	325 (21.9%)	69.6
A patient with *H. pylori* infection can stop the treatment once the symptoms improve	255 (17.2%)	258 (17.4%)	346 (23.3%)	420 (28.3%)	203 (13.7%)	59.2
Antibiotic resistance may make *H. pylori* treatment less effective	153 (10.3%)	200 (13.5%)	427 (28.8%)	472 (31.8%)	230 (15.5%)	65.7
Public awareness campaigns can reduce the spread of *H. pylori*-related diseases in the community	156 (10.5%)	163 (11.0%)	316 (21.3%)	536 (36.2%)	311 (21.0%)	69.2
Healthcare professionals often provide accurate information about *H. pylori* infection and its treatment	155 (10.5%)	203 (13.7%)	399 (26.9%)	488 (32.9%)	237 (16.0%)	66.1
Maintaining healthy habits reduces the risk of *H. pylori* infection	154 (10.4%)	161 (10.9%)	307 (20.7%)	551 (37.2%)	309 (20.9%)	69.4
Public awareness programs about *H. pylori* should be increased	174 (11.7%)	160 (10.8%)	315 (21.3%)	506 (34.1%)	327 (22.1%)	68.8
Ignoring digestive symptoms can lead to severe health problems	163 (11.0%)	175 (11.8%)	327 (22.1%)	508 (34.3%)	309 (20.9%)	68.4
I am concerned about the health risks and complications of *H. pylori* infection	153 (10.3%)	188 (12.7%)	357 (24.1%)	540 (36.4%)	244 (16.5%)	67.2
*H. pylori* infection is a common condition and does not require attention or follow-up	265 (17.9%)	258 (17.4%)	305 (20.6%)	470 (31.7%)	184 (12.4%)	59.3
Average attitude score	64.2%					

An average attitude was found concerning treatment effectiveness, where 56.7% of the participants (*n* = 840) strongly agreed/agreed that *H. pylori* infection can be completely cured when the correct treatment is followed. In relation to treatment behavior, only one third of the participants strongly disagreed/disagreed (*n* = 513, 34.6%) that the patients can stop treatment once symptoms improve. Concerning antibiotic resistance, 47.3% (*n* = 702) of the participants strongly agreed/agreed that resistance may reduce treatment effectiveness. Regarding public health campaigns, 57.2% (*n* = 847) of the participants strongly agreed/agreed that awareness campaigns can reduce the disease spread. In terms of trust in healthcare workers’ information, 48.9% (*n* = 725) of the participants strongly agreed/agreed that healthcare professionals provide accurate information about the disease (Table 3).

Concerning healthy habits, 58.1% of the participants (*n* = 860) strongly agreed/agreed that maintaining healthy habits reduces infection risk while about one-fifth of them strongly disagreed/disagreed (*n* = 315, 21.3%). For awareness programs, 56.2% (*n* = 833) of the participants strongly agreed/agreed that public awareness programs should be increased. On the other hand, 55.2% (*n* = 817) of the participants strongly agreed/agreed that ignoring digestive symptoms can lead to severe health problems and less than a quarter of them strongly disagreed/disagreed (*n* = 338, 22.8%). With respect to concerns about *H. pylori* complications, a little more than half of the participants (*n* = 784, 52.9%) strongly agreed/agreed that they are concerned about health risks and complications of this infection while less than a quarter of them strongly disagreed/disagreed (*n* = 341, 23.0%). Finally, 44.1% of the participants (*n* = 654) strongly agreed/agreed that *H. pylori* infection is a common condition that does not require attention while more than a third of them strongly disagreed/disagreed (*n* = 523, 35.3%; Table 3).

### 3.4 Assessment of the participants’ practices toward *H. pylori* infection

Table 4 presents the distribution of participants’ practices toward *H. pylori* infection. The practice score was calculated as described in the Methods section. The average practice score was 66.7%, indicating a moderate level of practice and the practice scores for the different practice items are shown on the far-right column. Concerning handwashing before eating, 61.9% of the participants (*n* = 918) reported always/often washing their hands while about one-fifth of them reported rarely/never doing so (*n* = 297, 20.1%). In terms of handwashing after using the restroom, 58.4% (*n* = 866) of the participants reported always/often washing their hands with about one-fifth of them reporting rarely/never doing so (*n* = 308, 20.8%). Concerning sharing utensils, more than half of the participants (54.7%; *n* = 811), reported they always/often avoid sharing eating or drinking utensils. For food safety, 55.3% of the participants (*n* = 820) reported always/often ensuring that their food is clean and well-cooked. With respect to water safety, 56.1% of the participants (*n* = 830) reported always/often ensuring that their drinking water is clean and safe while about a quarter of them reported rarely/never doing so (*n* = 367, 24.8%; Table 4).

**Table 4:** Frequency distribution of participants’ practice toward *H. pylori* infection.

Practice statement	Never	Rarely	Sometimes	Often	Always	Practice score
I wash my hands before eating	133 (9.0%)	164 (11.1%)	267 (18.0%)	469 (31.6%)	449 (30.3%)	72.6
I wash my hands after using the restroom	156 (10.5%)	152 (10.3%)	308 (20.8%)	337 (22.7%)	529 (35.7%)	72.6
I avoid sharing eating or drinking utensils with others	167 (11.3%)	189 (12.8%)	315 (21.3%)	351 (23.7%)	460 (31.0%)	70.1
I ensure the food I eat is clean and well-cooked	171 (11.5%)	180 (12.1%)	311 (21.0%)	329 (22.2%)	491 (33.1%)	70.6
I ensure the water I drink is clean and safe	172 (11.6%)	195 (13.2%)	285 (19.2%)	315 (21.3%)	515 (34.8%)	70.9
I get tested for *H. pylori* infection when I have digestive symptoms	223 (15.0%)	243 (16.4%)	361 (24.4%)	327 (22.1%)	328 (22.1%)	64.0
I visit a physician if I experience stomach pain, burning, or indigestion	183 (12.3%)	231 (15.6%)	402 (27.1%)	340 (22.9%)	326 (22.0%)	65.3
I complete the full course of antibiotics as prescribed by my physician	163 (11.0%)	198 (13.4%)	339 (22.9%)	364 (24.6%)	418 (28.2%)	69.1
I encourage my family members to get tested for *H. pylori* if someone in the household is infected	201 (13.6%)	222 (15.0%)	354 (23.9%)	353 (23.8%)	352 (23.8%)	65.8
I follow a healthy and safe diet to reduce the risk of *H. pylori* infection	208 (14.0%)	215 (14.5%)	355 (24.0%)	377 (25.4%)	327 (22.1%)	65.4
I eat food from restaurants and street vendors	216 (14.6%)	236 (15.9%)	406 (27.4%)	347 (23.4%)	277 (18.7%)	56.9
I wash my hands before preparing food	146 (9.9%)	169 (11.4%)	333 (22.5%)	323 (21.8%)	511 (34.5%)	71.9
I stop taking the antibiotic before completing the full treatment for *H. pylori* if my symptoms improve	282 (19.0%)	241 (16.3%)	361 (24.4%)	307 (20.7%)	291 (19.6%)	58.9
I drink filtered, treated, or bottled water instead of untreated water	173 (11.7%)	193 (13.0%)	355 (24.0%)	342 (23.1%)	419 (28.3%)	68.7
I self-medicate for stomach problems without consulting a doctor	242 (16.3%)	223 (15.0%)	413 (27.9%)	334 (22.5%)	270 (18.2%)	57.7
Practice average score	66.7%					

Regarding healthcare-seeking behavior, 44.2% of the participants (*n* = 655) reported always/often getting tested when experiencing digestive symptoms while less than a third of them reported rarely/never doing so (*n* = 466, 31.4%). Similarly, 44.9% of the participants (*n* = 666) reported always/often visiting a physician when experiencing gastrointestinal symptoms. With respect to treatment adherence, 52.8% of the participants (*n* = 782) reported always/often completing the full course of antibiotics while almost a quarter of them reported rarely/never doing so (*n* = 361, 24.4%). For encouraging family testing, less than half of the participants (*n* = 705, 47.6%) reported that they always/often encouraging family members to undergo testing. In terms of maintaining a healthy diet, 47.5% of the participants (*n* = 704) reported always/often following a healthy and safe diet while 28.5% (*n* = 423) of them reported rarely/never doing so (Table 4).

For consuming food from restaurants or street vendors, 42.1% of the participants (*n* = 624) reported always/often consuming such food. With respect to handwashing before food preparation, only 56.3% of the participants (*n* = 834) reported always/often washing their hands while about one-fifth of them reported rarely/never doing so (*n* = 315, 21.3%). Regarding early discontinuation of antibiotics, 40.3% of the participants (*n* = 598) reported that they always/often stop treatment before completion while more than a third of them reported they rarely/never do so (*n* = 523, 35.3%). For water consumption practices, about half of the participants (*n* = 761, 51.4%) reported that they always/often consume filtered or treated water. Finally, 40.7% of the participants (*n* = 604) reported always/often self-medicating behavior for stomach-related problems while less than a third of them reported rarely/never doing so (*n* = 465, 31.3%; Table 4). In summary, the above findings indicate low knowledge (55.5%), and moderate attitude (64.2%), and practice (66.7%) levels of the study participants.

### 3.5 Association between the participants’ knowledge regarding *H. pylori* infection and their sociodemographic characteristics

Table 5 presents the association between participants’ knowledge regarding *H. pylori* infection and their sociodemographic characteristics. The subgroup percentages reported in the text were derived from the corresponding cross-tabulations, whereas Table 5 presents only the statistical significance of the associations. For the knowledge that *H. pylori* infection is caused by bacterial infection, significant associations were observed with nationality (*p* < 0.001), employment status (*p* < 0.001), and level of education (*p* < 0.001). A higher proportion of Saudi participants (52.4%) were aware of the bacterial cause of the infection compared to non-Saudi participants (23.9%). Similarly, students and/or unemployed individuals (54.7%) were more likely to know the microbial cause of the infections compared to other employment categories. Also, those holding a bachelor’s degree (53.7%) were more knowledgeable of the bacterial cause of the infection compared to other educational level categories. Regarding the knowledge that *H. pylori* can be transmitted through contaminated food or water, significant associations were found with the region of residence (*p* = 0.023), monthly income (*p* = 0.031), and smoking status (*p* = 0.022). Residents of the Southern region had a higher knowledge score of this transmission route (69.1%) compared to other Saudi regions. Similarly, a higher proportion of participants who agreed with this statement was observed among participants earning <5,000 SAR (69.6%) compared to all other income categories. These are mainly students or unemployed participants. Also, regular smokers had a higher knowledge of this matter (74.8%) compared to other smoking categories. Concerning the statement that antibiotics alone are sufficient to treat *H. pylori* infection, a statistically significant association was observed only with the level of education (*p* = 0.006). Unexpectedly, a higher proportion of participants who disagreed with the statement was observed among those with less than a high school education (43.3%) compared to all other educational categories. Regarding the knowledge that *H. pylori* infection can cause peptic ulcer disease, a significant association was observed only with smoking status (*p* = 0.009). A higher proportion of ex-smoking participants agreed with that statement (77.3%) compared to other smoking categories. Regarding the knowledge that drinking unsafe or untreated water can cause *H. pylori* infection, significant associations were noted with gender (*p* = 0.001) and employment status (*p* = 0.049). In this regard, a slightly higher proportion of male participants were more knowledgeable about this issue (61.5%) compared to females (60.4%). Similarly, students/unemployed participants had a higher knowledge of this issue (64.0%) compared to other employment categories (Table 5 and unpublished cross-tabulations).

**Table 5:** Distribution of responses to knowledge questions regarding *H. pylori* and their association with sociodemographic characteristics.*

Knowledge statement	Age (years)	Gender	Marital status	Nationality	Region of residence	Employment Status	Level of education	Monthly income	Smoking status
*H. pylori* infection is caused by bacterial infection	0.447	0.071	0.208	**<0.001**	0.678	**<0.001**	**<0.001**	0.451	0.061
*H. pylori* can be transmitted through contaminated food or water	0.384	0.998	0.830	0.840	**0.023**	0.570	0.497	**0.031**	**0.022**
Antibiotics alone are sufficient to treat *H. pylori* infection	0.086	0.423	0.238	0.118	0.213	0.238	**0.006**	0.571	0.264
*H. pylori* infection can cause peptic ulcer disease	0.371	0.477	0.797	0.812	0.955	0.309	0.282	0.419	**0.009**
Drinking unsafe or untreated water can cause *H. pylori* infection	0.405	**0.001**	0.071	0.343	0.401	**0.049**	0.435	0.253	0.438
*H. pylori* infection is treated by antacids only	0.181	0.167	0.800	0.065	0.177	0.765	0.187	0.059	0.147
*H. pylori* infection can be diagnosed using non-invasive tests such as the urea breath test or stool antigen test	0.552	0.141	**0.014**	0.985	0.172	0.336	0.993	0.252	0.583
Washing hands before eating helps prevent *H. pylori* infection	0.906	0.628	0.054	0.804	**0.016**	**0.036**	0.560	**0.015**	**0.036**
Living with someone who has *H. pylori* increases the risk of infection	0.741	0.190	0.930	0.246	0.923	0.733	0.379	0.122	0.092
A common symptom of *H. pylori* infection is heartburn or pain in the upper abdomen	0.554	0.082	**0.032**	0.242	**0.030**	0.618	0.096	0.575	**0.026**
Long-term *H. pylori* infection can lead to stomach (gastric) cancer	0.347	0.468	0.810	0.393	0.427	0.177	0.284	0.155	**0.004**
The breath test is one of the most accurate tests for diagnosing *H. pylori* infection	0.423	0.135	0.461	0.301	0.077	0.107	0.629	0.382	0.104
Completing the full course of treatment is important for *H. pylori* eradication	0.721	0.103	0.129	0.294	**0.003**	0.298	**0.036**	0.205	0.208
Consuming fast food frequently may increase the risk of *H. pylori* infection	0.116	0.349	**0.006**	0.419	0.477	0.291	0.344	0.171	0.466
Washing fruits and vegetables well helps prevent *H. pylori* infection	0.796	0.263	**0.001**	0.369	0.158	**0.022**	0.127	0.382	0.058

*Chi square test p values are shown. Bold = Statistically significant.

In terms of knowing that washing hands before eating helps prevent *H. pylori* infection, significant associations were observed with the region of residence (*p* = 0.016), employment status (*p* = 0.036), monthly income (*p* = 0.015), and smoking status (*p* = 0.036). A higher proportion of participants who agreed with this statement was observed among residents of the Western region (68.9%) compared to other Saudi regions. Similarly, a higher proportion of participants who are aware of that statement was observed among retired/volunteering participants (73.7%) compared to other employment categories. Also, those earning <5,000 SAR were more knowledgeable of this matter (70.4%) compared to other income categories. In addition, a higher knowledge score of this issue was observed among ex-smokers (69.3%) compared to other smoking categories. Regarding the knowledge that long-term *H. pylori* infection can lead to gastric cancer, a significant association was, also, observed with smoking status (*p* = 0.004). In this regard, a higher knowledge score was observed among social smokers (65.2%) compared to other smoking categories (Table 5 and unpublished cross-tabulations).

Concerning the knowledge that completing the full course of treatment is important, statistically significant associations were observed with the region of residence (*p* = 0.003) and the level of education (*p* = 0.036). In this regard, a higher knowledge score was observed among residents of both the Central and Southern regions (65.5% each) compared to other Saudi regions. Additionally, participants holding a Master’s or Doctorate degree had more awareness (70.2%) compared to other educational level categories. Other remaining significant knowledge associations are presented in Table 5 while no statistically significant associations were observed between all demographic variables and the statements regarding treatment with antacids alone sufficiency for treatment, living with an infected individual, and the accuracy of the breath test (Table 5 and unpublished cross-tabulations).

### 3.6 Association between the participants’ attitudes toward *H. pylori* infection and their sociodemographic characteristics

Table 6 presents the association between participants’ attitudes toward *H. pylori* infection and their sociodemographic characteristics. The subgroup percentages reported in the text were derived from the corresponding cross-tabulations, whereas Table 6 presents only the statistical significance of the associations. For the statement that *H. pylori* infection is a major public health issue in Saudi Arabia, significant associations were observed with age (*p* = 0.002), marital status (*p* = 0.013), monthly income (*p* = 0.001), and the smoking status (*p* < 0.001). Participants aged 18–30 years were more likely to strongly agree/agree with the statement that *H. pylori* infection is a major public health issue in Saudi Arabia (with only 18.0% proper attitude) compared to other age categories that had lower proper attitude percentages. Similarly, divorced participants (38.1%), participants earning >10,000 SAR and those earning <5,000 SAR (both 17.5%), and ex-smokers (29.6%) were more likely to strongly agree/agree with that statement compared to others with the respective demographic categories. Concerning the statement that *H. pylori* infection is a serious health issue that requires professional medical attention, significant associations were observed with gender (*p* = 0.008), monthly income (*p* = 0.006), and smoking status (*p* = 0.016). Female participants were more likely to strongly agree/agree with this statement (59.0%) compared to males (52.1%). Similarly, participants earning <5,000 SAR (57.1%) and regular smokers (62.6%) were more likely to agree with that statement compared to others with parallel demographic categories. In terms of the statement that maintaining good hand hygiene and safe food practices can prevent *H. pylori* infection, a significant association was observed only with nationality (*p* = 0.017). In this respect, non-Saudi participants had a slightly higher tendency to strongly agree/agree with this statement (56.4%) compared to Saudi participants (55.8%; Table 6 and unpublished cross-tabulations).

**Table 6:** Distribution of attitudes toward *H. pylori* infection and their association with sociodemographic characteristics.*

Attitude statement	Age (years)	Gender	Marital status	Nationality	Residence area	Employment status	Level of education	Monthly income	Smoking status
*H. pylori* infection is a major public health issue in Saudi Arabia	**0.002**	0.155	**0.013**	0.237	0.158	0.088	0.483	**0.001**	**<0.001**
Preventing *H. pylori* infection is not important for maintaining good digestive health	**0.032**	0.562	0.766	0.604	0.581	**0.022**	0.307	**0.018**	0.67
*H. pylori* infection is a serious health issue that requires professional medical attention	0.127	**0.008**	0.178	0.656	0.926	0.609	0.194	**0.006**	**0.016**
Regular screening for *H. pylori* is unnecessary and does not contribute to the prevention of gastric cancer	0.376	0.177	**0.019**	0.178	**0.024**	**0.029**	**0.018**	**0.032**	0.124
Maintaining good hand hygiene and safe food practices can prevent *H. pylori* infection	0.259	0.346	0.355	**0.017**	0.084	0.427	0.258	0.158	0.716
*H. pylori* infection can be completely cured when the correct treatment is followed	0.416	**0.015**	**0.022**	**0.005**	0.119	0.289	0.108	0.62	0.963
A patient with *H. pylori* infection can stop the treatment once the symptoms improve	0.9	0.07	0.069	**0.029**	**0.04**	0.059	0.15	**0.001**	**0.005**
Antibiotic resistance may make *H. pylori* treatment less effective	0.975	0.286	0.268	0.577	0.239	**0.019**	**0.019**	0.095	**0.01**
Public awareness campaigns can reduce the spread of *H. pylori*-related diseases in the community	0.4	0.126	0.181	**0.026**	0.071	0.671	0.667	0.522	0.479
Healthcare professionals often provide accurate information about *H. pylori* infection and its treatment	0.811	0.834	0.22	0.099	**0.032**	0.629	0.19	**0.002**	0.567
Maintaining healthy habits reduces the risk of *H. pylori* infection	0.488	0.753	0.261	0.842	**0.047**	0.342	0.172	0.147	0.153
Public awareness programs about *H. pylori* should be increased	0.732	0.343	**0.036**	0.794	**0.019**	0.083	0.5	0.189	0.069
Ignoring digestive symptoms can lead to severe health problems	0.326	0.258	**0.007**	0.164	0.504	0.206	0.735	0.069	0.463
I am concerned about the health risks and complications of *H. pylori* infection	0.053	0.47	0.147	0.774	0.058	0.541	0.066	0.247	0.094
*H. pylori* infection is a common condition and does not require attention or follow-up	0.283	0.373	**0.021**	**0.017**	**0.001**	**0.008**	0.233	**0.004**	0.07

^*^Chi square test *p*-values are shown. Bold = Statistically significant.

Regarding the statement that *H. pylori* infection can be completely cured when the correct treatment regimen is followed, significant associations were observed with gender (*p* = 0.015), marital status (*p* = 0.022), and nationality (*p* = 0.005). Female participants were more likely to agree with this statement (59.8%) compared to males (54.2%). Similarly, widowed (69.7%) and Saudi participants (57.4%) were more likely to strongly agree/agree with this statement compared to others with parallel demographic categories. Concerning the statement that a patient with *H. pylori* infection can stop the treatment once the symptoms improve, significant associations were observed with nationality (*p* = 0.029), region of residence (*p* = 0.040), monthly income (*p* = 0.001), and smoking status (*p* = 0.005). Saudi participants were more likely to strongly disagree/disagree with this statement (35.6%) compared to non-Saudi participants (23.0%) indicating low proper attitudes. Similarly, participants residing in the Western region (38.0%), participants earning >10,000 SAR (38.0%), and ex-smokers (39.7%) were more likely to strongly disagree/disagree with that statement compared to others with parallel demographic categories. For the statement that antibiotic resistance may make *H. pylori* treatment less effective, significant associations were observed with employment status (*p* = 0.019), level of education (*p* = 0.019), and smoking status (*p* = 0.010). Self-employed participants were more likely to strongly agree/agree with this statement (53.9%) compared to other employment categories. Similarly, participants with a higher diploma degree (57.3%) and non-smokers (48.2%) were more likely to strongly agree/agree with that statement compared to others with parallel demographic categories (Table 6 and unpublished cross-tabulations).

Concerning the statement that ignoring digestive symptoms can lead to severe health problems, a significant association was observed only with marital status (*p* = 0.007). Divorced participants were more likely to strongly agree/agree with this statement (76.2%) compared to other marital status categories. For the statement that *H. pylori* infection is a common condition that does not require attention or follow-up, significant associations were observed with marital status (*p* = 0.021), nationality (*p* = 0.017), the region of residence (*p* = 0.001), employment status (*p* = 0.008), and the monthly income (*p* = 0.004). In this regard, divorced participants were more likely to strongly disagree/disagree with this statement (42.8%) compared to other marital status categories. Also, Saudi participants were more likely to strongly disagree/disagree with that statement (36.3%) compared to non-Saudi participants (23.1%). In addition, participants from the Western region were more likely to strongly disagree/disagree with that statement (38.8%) compared to other Saudi regions. Moreover, retired/volunteering participants were more likely to strongly disagree/disagree with that statement (43.8%) compared to other employment categories. Furthermore, participants earning <5,000 SAR monthly were more likely to strongly disagree/disagree with that statement (40.5%) compared to other income groups. Other remaining significant attitude associations are presented in Table 6 while no significant associations were observed regarding concern about the health risks and complications of *H. pylori* infection and various demographic characteristics (Table 6 and unpublished cross-tabulations).

### 3.7 Association between the participants’ practice regarding *H. pylori* infection and their sociodemographic characteristics

Table 7 shows the association between the participants’ practices and their sociodemographic characteristics. The subgroup percentages reported in the text below were derived from the corresponding cross-tabulations, whereas Table 7 presents only the statistical significance of the associations. For the practice of washing hands before eating, significant associations were observed with gender (*p* = 0.041), nationality (*p* = 0.035), employment status (*p* < 0.001), level of education (*p* = 0.006), and average monthly income (*p* = 0.001). Female participants were more likely to behave appropriately regarding handwashing before eating (63.4%) compared to males (60.7%). Similarly, Saudi participants reported more appropriate handwashing practices (62.2%) compared to non-Saudi participants (58.1%). Regarding employment status, private sector employees were more likely to report appropriate handwashing practices (64.2%) compared to other employment categories. Also, participants holding a higher diploma degree reported more appropriate hand-washing practices (70.8%) compared to other educational categories. Additionally, participants earning <5,000 SAR reported higher appropriate hand-washing practices (66.7%) compared to other income groups. For the practice of avoiding sharing eating or drinking utensils with others, significant associations were observed with gender (*p* = 0.013), marital status (*p* = 0.019), the region of residence (*p* = 0.039), and employment status (*p* = 0.005). Female participants behaved more appropriately regarding avoidance of sharing eating or drinking utensils (56.0%) compared to males (53.7%). Similarly, divorced participants reported more appropriate practices (57.1%) compared to other marital status categories. Also, participants living in the Western region reported higher appropriate practices (57.2%) compared to other regional categories. Additionally, government employees reported more appropriate practices (58.1%) compared to other employment categories. Concerning the practice of ensuring that food is clean and well-cooked, significant associations were observed with age (*p* = 0.019) and employment status (*p* = 0.012). Participants aged 18–30 years and 41–50 years behaved more appropriately regarding food cleanliness and cooking practices (57.4% each) compared to other age groups. Similarly, government employees reported more appropriate practices (57.8%) compared to other employment categories. Regarding the practice of ensuring that drinking water is clean and safe, a significant association was observed only with employment status (*p* = 0.017) with government employees behaving more appropriately (57.4%) compared to other employment categories (Table 7 and unpublished cross-tabulations).

**Table 7:** Distribution of practices toward *H. pylori* infection and their association with sociodemographic characteristics.

Practice statement	Age (years)	Gender	Marital status	Nationality	Region of residence	Employment status	Level of education	Average monthly income	Smoking status
I wash my hands before eating	0.195	**0.041**	0.148	**0.035**	0.059	**<0.001**	**0.006**	**0.001**	0.142
I wash my hands after using the restroom	0.06	0.425	**<0.001**	0.079	**0.007**	**0.001**	**0.025**	**0.039**	0.136
I avoid sharing eating or drinking utensils with others	0.393	**0.013**	**0.019**	0.063	**0.039**	**0.005**	0.386	0.705	0.223
I ensure the food I eat is clean and well-cooked	**0.019**	0.579	0.521	0.4	0.176	**0.012**	0.806	0.209	0.267
I ensure the water I drink is clean and safe	0.428	0.528	0.11	0.311	0.083	**0.017**	0.277	0.135	0.17
I get tested for *H. pylori* infection when I have digestive symptoms	0.423	0.453	**0.008**	0.137	0.241	**0.002**	0.359	0.068	0.112
I visit a physician if I experience stomach pain, burning, or indigestion	0.072	0.429	0.749	0.317	**0.008**	0.11	0.797	0.409	0.143
I complete the full course of antibiotics as prescribed by my physician	0.105	0.076	0.268	0.745	0.8	0.457	0.909	0.674	0.595
I encourage my family members to get tested for *H. pylori* if someone in the household is infected	0.739	0.587	0.085	0.113	**0.034**	**0.031**	0.472	0.314	0.084
I follow a healthy and safe diet to reduce the risk of *H. pylori* infection	0.435	0.409	0.734	**0.012**	0.339	0.111	0.959	0.976	0.283
I eat food from restaurants and street vendors	0.272	0.182	0.248	0.088	0.427	0.496	0.248	**0.032**	0.831
I wash my hands before preparing food	0.061	0.135	0.299	**0.007**	0.107	**<0.001**	0.219	0.067	**<0.001**
I stop taking the antibiotic before completing the full treatment for *H. pylori* if my symptoms improve	0.11	0.517	0.313	0.215	**0.018**	0.129	0.049	0.119	**0.01**
I drink filtered, treated, or bottled water instead of untreated water	**0.03**	0.933	**0.027**	0.057	**0.019**	0.149	0.517	0.33	0.288
I self-medicate for stomach problems without consulting a doctor	0.237	0.951	0.216	0.754	0.784	0.242	0.271	**0.01**	0.155

^*^ Chi square test *p*-values are shown. Bold = Statistically significant.

For the practice of getting tested for *H. pylori* infection when experiencing digestive symptoms, significant associations were observed with marital (*p* = 0.008) and employment states (*p* = 0.002). Widowed participants reported higher appropriate testing practices (60.6%) compared to other marital categories. Similarly, part-time employees reported higher appropriate practices (57.3%) compared to other employment categories. Regarding the practice of visiting a physician when experiencing stomach pain, burning, or indigestion, a significant association was observed only with the region of residence (*p* = 0.008). Participants living in the Northern region behaved more appropriately regarding seeking medical care for digestive symptoms (50.5%) compared to other regional categories. In terms of the practice of following a healthy and safe diet to reduce the risk of *H. pylori* infection, a significant association was observed only with nationality (*p* = 0.012). In this regard, Saudi participants behaved more appropriately regarding healthy dietary practices (48.1%) compared to non-Saudi participants (41%). For the practice of washing hands before preparing food, significant associations were observed with nationality (*p* = 0.007), employment status (*p* < 0.001), and smoking status (*p* < 0.001). Saudi participants behaved more appropriately regarding hand washing before food preparation (57.4%) compared to non-Saudi participants (43.6%). Similarly, Student/unemployed participants reported the most appropriate practices (58.0%) compared to other employment categories. Also, ex-smokers reported more appropriate practices (56.8%) compared to other smoking categories. Concerning the practice of not stopping antibiotic treatment before completing the full course despite symptom improvement, significant associations were observed with the region of residence (*p* = 0.018), level of education (*p* = 0.049), and smoking status (*p* = 0.010). In this regard, participants living in the Western region behaved more appropriately regarding completion of antibiotic treatment (39.3%) compared to other regional categories. Similarly, participants holding a master’s degree or Doctorate reported more appropriate practices (41.2%) compared to other educational categories. Also, ex-smokers reported more appropriate practices (48.9%) compared to other smoking categories. For the practice of drinking filtered, treated, or bottled water instead of untreated water, significant associations were observed with age (*p* = 0.030), marital status (*p* = 0.027), and the region of residence (*p* = 0.019). Participants aged 41–50 years behaved more appropriately (54.8%) compared to other age groups. Similarly, married participants reported more appropriate practices (53.2%) compared to other marital categories. Also, participants living in the Central region reported more appropriate practices (58.3%) compared to other regional categories. Regarding the practice of not self-medicating for stomach problems without consulting a physician, a significant association was observed only with the average monthly income (*p* = 0.010). In this respect, participants earning >10,000 SAR were more likely to behave appropriately regarding avoidance of self-medication (36.3%) compared to other income groups. Other remaining significant practice associations are presented in Table 7 while no significant associations were found between any of the sociodemographic variables and the practice of completing the full course of antibiotics as prescribed by the physician (Table 7 and unpublished cross tabulations).

## 4 Discussion

This nationwide study assessed the KAP levels among 1,482 Saudi residents regarding *H. pylori* infection and its complications and management. The mean KAP scores among the general public of the Kingdom were 55.5, 64.2, and 66.7%, respectively, indicating low knowledge and moderate attitude and practice levels. Item-level analysis revealed that awareness was uneven. Knowledge of basic preventive measures was comparatively stronger, with 66.9% correctly identifying handwashing as a preventive practice and 64.8% recognizing contaminated food or water as a transmission routes, while treatment-related knowledge was notably weaker, with only 30.8% correctly identifying that antibiotics alone are not sufficient for treatment, and only 29.4% correctly recognizing that antacids alone cannot eradicate the infection. Attitudes were generally favorable toward the seriousness of the infection and the value of treatment adherence, though the lowest attitude score (42.8%) corresponded to recognition of *H. pylori* as a major public health issue in Saudi Arabia. Practice scores demonstrated greater strength in hygiene-related behaviors than in treatment- and healthcare-seeking behaviors. Several aspects of these findings warrant further discussion.

First, within the knowledge domain, the current findings align with a broader body of literature that has documented consistently limited public knowledge about *H. pylori*, especially regarding transmission and its link to gastric cancer (16). For instance, a study of 808 patients in Riyadh reported that 36.9% of the participants either incorrectly believed that *H. pylori* infection does not cause indigestion or reported that they did not know (4), which is analogous to our 37% rate. Comparably, some Saudi studies have consistently reported uneven *H. pylori* awareness, characterized by relatively stronger recognition of preventive behaviors alongside persistent misconceptions regarding transmission routes, disease complications, and diagnostic approaches (20, 21). Our findings also align with this pattern, particularly in relation to the limited understanding of treatment-related concepts despite comparatively better preventive knowledge. In this respect, the most pronounced gap was in treatment-related issues, where only 30.8 and 29.4% of the participants correctly identified that antibiotic or antacids monotherapy is insufficient for infection eradication, respectively. Most of the participants either responded incorrectly or reported uncertainty about these two items. By contrast, 63.8% of the participants correctly recognized the importance of completing the full treatment course. This pattern suggests that while participants were aware of the need for treatment completion in principle, the specific components of eradication therapy were poorly understood. Likewise, a more pronounced knowledge deficit was observed among university students in a study from Riyadh, where <10% of them demonstrated sufficient understanding of transmission, complications, or prevention (5), although direct comparison with our current findings is limited by differences in sampling frames and participants’ demographic characteristics. Regional surveys further demonstrate that awareness of the association between *H. pylori* infection and gastric cancer remains limited. In Al-Ahsa (Saudi Arabia), although 54.9% of the respondents achieved a good overall awareness, only 24.2% of the participants recognized gastric cancer as a possible complication of this infection (22). Likewise, a study from Hail (Saudi Arabia), found that only 23.1% of the participants demonstrated good knowledge, with particularly poor recognition of gastric cancer-related complications (23). Comparable international findings have also been observed (8, 12, 14, 24, 25). For example, similar misconceptions regarding symptoms, transmission, and disease complications have also been reported in Pakistan despite increasing public exposure to health information (24). Also, in Bosnia and Herzegovina, 58.5% of the participants provided mostly correct responses overall, yet knowledge related to testing and treatment remained limited (25). On the other hand, a nationwide Japanese study reported that although the public awareness of *H. pylori* was high, detailed understanding of its characteristics, testing, and treatment methods remained substantially low (13), mirroring the treatment-and diagnosis-related knowledge gaps identified in our study.

Second, within the attitude domain, a notable discrepancy was observed between population-level and personal risk perception, with 63.2% of the participants strongly disagreeing/ disagreeing that *H. pylori* represent a major public health concern in Saudi Arabia, while 52.9% simultaneously reported personal concern regarding associated health risks. Overall, attitudes were moderate although there were some inconsistencies. For instance, 55.8% of the participants acknowledged the importance of hand hygiene and safe food practices in prevention, and 56.7% of them believed that *H. pylori* can be completely cured with correct treatment, yet only 15.3% agreed that the infection is a major public health issue, and 42.0% endorsed the inappropriate belief of stopping treatment once symptoms improve. A further 44.1% agreed that *H. pylori* is a common condition not requiring follow-up, contradicting the expressed personal concern. Consistent with these patterns, one report (8), found suboptimal attitudes and practices among the Jordanian public despite the generally acceptable awareness levels. Also, another study in China (14), demonstrated that positive preventive attitudes can coexist with incomplete disease-specific knowledge, while showing that higher knowledge levels were associated with more appropriate attitudes and preventive behaviors. In addition, attitudes were similarly suboptimal in the United Arab Emirates, with only 33% of the respondents indicating willingness to seek medical help for ulcer symptoms (17), a reluctance that parallels the present study’s low recognition of *H. pylori* as a public health issue. By contrast, a Bosnian survey revealed a strong support for *H. pylori* screening and treatment, as 85.3% of the participants endorsed national screening programs (25), suggesting that attitudinal positivity may be shaped by differences in healthcare infrastructure and public education. A similar discrepancy between favorable attitudes and actual screening uptake was documented in Croatia, where despite relatively good knowledge and high support for screening (74.7%), only 27.9% had actually been tested (18). In the present study, the discrepancy between personal concerns regarding infection-related health risks and lower recognition of *H. pylori* as a public health issue may reflect variability in risk perception that was not further explored with the current design.

Third, within the practice domain, hygiene-related behaviors were more consistently reported than treatment-related behaviors. Hand washing before eating and after restroom usage were reported as always/often by 61.9 and 58.4% of the participants, respectively. By contrast, 40.3% of the participants reported always/often discontinuing antibiotic therapy upon symptoms’ improvement, and 40.7% reported always/often self-medicating for gastric complaints without medical consultation. Healthcare-seeking behaviors were also limited, with only 44.2% reporting that they always/often seek testing upon experiencing digestive symptoms. The stronger performance on hygiene-related practices relative to treatment-oriented behaviors aligns with earlier regional and international studies. Unhealthy dietary and lifestyle patterns may further compound these challenges. For example, while hygiene practices were generally acceptable in the United Arab Emirates, unhealthy dietary and lifestyle patterns, such as smoking, were also prevalent (17). Another Chinese survey found that eating out and group dining were associated with *H. pylori* infection, and 40.5% of the participants never used dedicated serving spoons or chopsticks; instead, they used their personal utensils to take food from shared dishes, a habit that facilitates person-to-person transmission (19). Such behaviors could contribute to the persistence of infection in the community, although they were not specifically measured in the current study. Treatment non-adherence has been recognized as a widespread problem, with side effects and financial barriers cited as contributing factors (12), but these barriers were not assessed in the present study and their relevance to this sample remains undetermined. Additionally, a survey in Cameroon (11), reported considerable variability in diagnostic and treatment practices among primary care physicians, including inconsistent eradication follow-up and empiric treatment approaches. Diagnostic uncertainty and empirical treatment without prior confirmation have also been noted among Turkish general practitioners (10), indicating that gaps in the general population’s knowledge may be mirrored and compounded by suboptimal physician practices. Comparable variability in awareness and management-related knowledge has been reported among medical students and physicians in Saudi Arabia (6, 7), suggesting that inconsistencies related to *H. pylori* management may extend beyond the general population. Recent data from primary care centers in Riyadh further illustrate this point. In this respect, although most of the general practitioners managed *H. pylori* cases, only 59.3% of them adhered to the recommended 14-day treatment duration, and the regimen choice varied significantly with experience and practice setting (26).

Fourth, the sequential structure of the KAP framework suggests that knowledge may influence attitudes, which in turn can shape practices. However, the findings of this study only partially align with this theoretical model. The observed knowledge deficit, particularly in treatment-related items where fewer than one-third of the participants correctly identified that antibiotics monotherapy or antacids alone are insufficient for *H. pylori* eradication, may suggest a possible relationship with the relatively high proportion of participants reporting premature discontinuation of antibiotic therapy (40.3%). Similarly, the tendency toward stopping treatment upon symptoms’ improvement may reflect underlying gaps in treatment-related knowledge, although these relationships cannot be confirmed due to the cross-sectional design of this study. Despite the relatively favorable attitudes toward hygiene and treatment adherence, several potentially inappropriate practices remained common, particularly premature discontinuation of antibiotic therapy and self-medication without medical consultation. The practice domain showed that a substantial proportion of the participants engaged in early discontinuation of therapy, indicating a possible partial inconsistency between knowledge, attitudes, and practices in this population rather than a complete disconnect. Notably, the mean practice score (66.7%) slightly exceeded the mean attitude score (64.2%), suggesting that the KAP relationship may not be strictly linear in this setting, a pattern that has been reported previously, where certain preventive behaviors are influenced by habitual or externally reinforced practices rather than knowledge alone (18, 19). However, causal relationships between these domains cannot be established, and future studies using longitudinal or advanced analytical methods are needed to better clarify these pathways.

Fifth, significant associations between the different KAP items and sociodemographic variables were observed across multiple KAP domains, though these were inconsistent in pattern and scope. Nationality was significantly associated with the correct identification of *H. pylori* as a bacterial infection and with several other attitude items. Also, educational level was significantly associated with specific knowledge items, including antibiotics alone as sufficient for treatment and treatment completion, though the direction of association was not uniformly positive. Oddly, for the antibiotic sufficiency, participants with less than a high school education demonstrated a higher awareness rate (43.3%) among all educational categories. Additionally, the region of residence was significantly associated with selected knowledge and practice items; albeit inconsistent; including the importance of treatment completion and early antibiotic discontinuation. Moreover, monthly income was associated with specific attitude items, including perception of infection seriousness and treatment-stopping behavior. Furthermore, gender was significantly associated with fewer items overall, reaching significance in relation to knowledge of water-borne transmission and attitudes toward infection seriousness. These demographic differences echo patterns were reported in the literature, where socioeconomic status, educational background, and access to medical information have been linked to variations in awareness and preventive behaviors. Similar associations were documented in a Saudi population-based study (15), and females in Hail who heard of the infection had higher knowledge scores (23). Likewise, a Bosnian study identified female sex, higher education, and prior screening as predictors of better *H. pylori* knowledge (25). By contrast, an investigation in Al-Baha on gastric cancer awareness found that males had higher knowledge of risk factors (27), suggesting that the direction of gender-based differences may depend on the disease context and the population sampled. Furthermore, a Croatian study noted that older age, male sex, and rural residence were linked to lower knowledge levels (18). These associations, reported herein, should be interpreted with caution, as the large number of chi-square comparisons performed across 15 items and up to nine demographic variables per domain was not adjusted for multiple testing, increasing the probability of type I error. Also, effect sizes were not reported, and statistical significance at this sample size (*n* = 1,482) does not necessarily indicate practical relevance.

Finally, compared with many previous regional studies, the present study incorporated broader geographic representation and more detailed evaluation of treatment-related misconceptions and healthcare-seeking behaviors, allowing a more nuanced assessment of *H. pylori*-related awareness and practices among the Saudi population. Although this study has several strengths, including the large sample size, inclusion of many participants from all regions of the Kingdom, and a detailed item-level assessment across multiple KAP domains related to *H. pylori* infection, it has several limitations that should be considered when interpreting our findings. The use of a non-probability web-based convenience sampling approach may have introduced selection bias by favoring individuals with internet access and active social media use, potentially leading to the over representation of younger and more educated participants. Also, all measures were self-reported, introducing potential social desirability bias, particularly for treatment-related behaviors. In addition, the cross-sectional design precludes causal inference between variables. Furthermore, we did not ask if the participant her/himself was infected with *H. pylori*. Taken together, the findings may not be fully generalizable to the overall Saudi population and may underrepresent certain groups.

## 5 Conclusion

This cross-sectional study of 1,482 Saudi residents identified low knowledge and moderate attitudes and practices regarding *H. pylori* infection, with mean scores of 55.5, 64.2, and 66.7%, respectively. Notable gaps were observed in treatment-related knowledge and selected health behaviors. Knowledge of eradication therapy was low. On the other hand, awareness of basic preventive measures was comparatively high, while attitude findings indicated low recognition of *H. pylori* as a public health concern. Within the practice domain, the lowest scores were observed for self-medication and early discontinuation of antibiotic therapy. Besides, variation in KAP responses was observed across sociodemographic groups. These findings support targeted health education initiatives aimed at addressing misconceptions related to eradication therapy and antibiotic adherence. However, the underlying determinants of these knowledge and practice gaps were not assessed in this study. Further studies are, therefore, needed to identify these determinants and to evaluate the effectiveness of interventions using appropriately designed, population-representative studies.

## Data Availability

The original contributions presented in the study are included in the article/supplementary material, further inquiries can be directed to the corresponding author.
